# HEAD Metamodel: Hierarchical, Extensible, Advanced, and Dynamic Access Control Metamodel for Dynamic and Heterogeneous Structures

**DOI:** 10.3390/s21196507

**Published:** 2021-09-29

**Authors:** Nadine Kashmar, Mehdi Adda, Hussein Ibrahim

**Affiliations:** 1 Département de Mathématiques, Informatique et Génie, Université du Québec à Rimouski, 300 Allée des Ursulines, Rimouski, QC G5L 3A1, Canada; mehdi_adda@uqar.ca; 2 Institut Technologique de Maintenance Industrielle, 175 Rue de la Vérendrye, Sept-Îles, QC G4R 5B7, Canada; hussein.ibrahim@itmi.ca

**Keywords:** access control, metamodel, policy, hierarchy, security and privacy, IoT, Industry 4.0, heterogeneous

## Abstract

The substantial advancements in information technologies have brought unprecedented concepts and challenges to provide solutions and integrate advanced and self-ruling systems in critical and heterogeneous structures. The new generation of networking environments (e.g., the Internet of Things (IoT), cloud computing, etc.) are dynamic and ever-evolving environments. They are composed of various private and public networks, where all resources are distributed and accessed from everywhere. Protecting resources by controlling access to them is a complicated task, especially with the presence of cybercriminals and cyberattacks. What makes this reality also challenging is the diversity and the heterogeneity of access control (AC) models, which are implemented and integrated with a countless number of information systems. The evolution of ubiquitous computing, especially the concept of Industry 4.0 and IoT applications, imposes the need to enhance AC methods since the traditional methods are not able to answer the increasing demand for privacy and security standards. To address this issue, we propose a Hierarchical, Extensible, Advanced, and Dynamic (HEAD) AC metamodel for dynamic and heterogeneous structures that is able to encompass the heterogeneity of the existing AC models. Various AC models can be derived, and different static and dynamic AC policies can be generated using its components. We use Eclipse (xtext) to define the grammar of our AC metamodel. We illustrate our approach with several successful instantiations for various models and hybrid models. Additionally, we provide some examples to show how some of the derived models can be implemented to generate AC policies.

## 1. Introduction

The current generation of networking environments, referring to dynamic and ever-evolving environments, such as the Internet of Things (IoT), cloud computing, etc., with several millions of users who need access to information stored in distributed data centers and servers via various types of devices (wearable devices, mobile phones, tablets, …), makes the process of controlling access challenging and very complicated. Moreover, the emergence of ubiquitous computing with heterogeneous devices, platforms, etc., especially the concept of Industry 4.0 and IoT applications, releases new prospects to traditional information systems and access control (AC) methods by merging new technologies and services for seamless access to information sources at anytime and anywhere [1,2,3]. This reality, in addition to the heterogeneity of the AC models that are implemented in the different centralized and distributed computing environments, makes the process of controlling access even more complicated, as the method of AC should answer the needs of any computing environment by including the heterogeneity of AC models, and being upgradable and dynamic to follow possible technology progressions [4,5].

Moreover, AC policies are among the most significant security mechanisms that are essential to increase the privacy and confidence of an information system. Up to the present time, AC research and real-world AC implementations to define and enforce AC policies broadly fall under one of the five stages:

1. Traditional AC models discretionary access control (DAC), mandatory access control (MAC), role-based access control (RBAC), attribute-based access control (ABAC) [4,6];
2. Hybrid models, by means of combining features of two or more AC models, for example, hybrid RBAC/ABAC model [7];
3. Extended AC models, by means of adding new component(s) to a model to enhance its features, for example, [8];
4. Abstract AC models, by means of abstracting a model and adding new components to it, then deriving different instances of it, for example, [9];
5. AC metamodels, by means of including all of the above, for example, [10].

**Figure 1:**
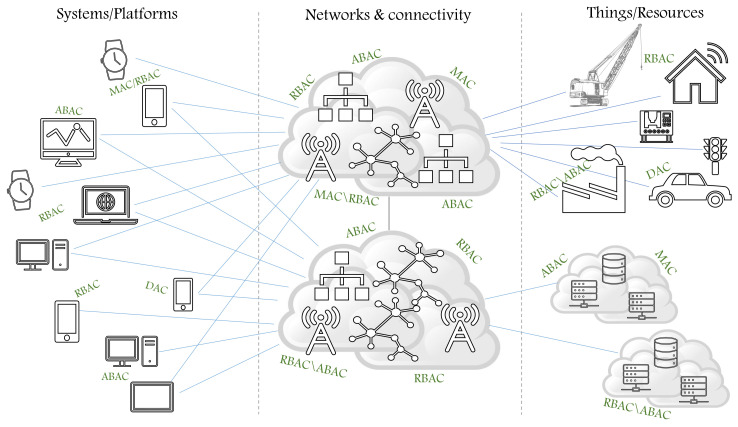
The dynamic and heterogeneous structures.

In the literature, different AC models are implemented to define and enforce AC policies in order to specify users’ access rights to resources and verify that they can only access resources they are allowed to in a given context. In Figure 1, we illustrate the notion of heterogeneous structures, which include heterogeneous systems, platforms, networks, and devices, in addition to the heterogeneity of the implemented AC models to define and enforce different AC policies, such as password policy, network access policy, remote access policy, etc. With the evolution of technology trends, it is realized that traditional models, hybrid models, extended AC models, and abstract AC models no longer meet the increasing demand of privacy and security standards; in other words, they are currently insufficient to handle all the AC requirements [5]. To enhance AC methods, the era of developing AC metamodels began within the decade to serve as unifying frameworks with advanced AC features that are able to include most features of AC models in order to define a larger set of AC policies and upgrade the defined policies [5,10,11]. The AC metamodel should allow security experts and system administrators to create the needed components to define/upgrade any static and dynamic AC policy since controlling users’ access and the actions they perform on information cannot be ignored when developing strategies related to information security [6,12]. An information system (IS) must follow up the evolution of security threats by designing and including modern security concepts and practices and incorporating them with the information system development life cycle (SDL). With the evolution of SDL, various studies have focused on the importance of collaboration and communication between software operators and developers. Recently, the need for security prompted the collaboration between developers and operators by involving security experts from the start of SDL [13,14].

However, implementing AC methods in complicated and distributed environments with several millions of users who might be assigned to different levels of roles, categories, groups, etc., and who request access to millions of objects, which might be distributed also in levels in several sites, need a generic, dynamic, and extensible AC metamodel that supports the hierarchy of components, for example, objects, roles, categories, actions, and maybe conditions. Unfortunately, despite the existing metamodels tackling specific issues related to certain computing environments, they lack some essential features and have several limitations (summarized in Section 2) [5,6,11]. Our concern in this paper falls under the fifth stage of developing AC methods. For this purpose, we propose a Hierarchical, Extensible, Advanced, and Dynamic (HEAD) AC metamodel with unconventional features to assist developers and security experts to include its components in designing secure ISs that conform to organizational AC security policies. In this paper, we tackle the generic, dynamic, extensible, and hierarchical limitations of the existing metamodels. We propose the kernel elements of the HEAD metamodel (a preliminary and general instance of our metamodel is presented in [10]); we unify the concepts of the heterogeneous AC components and include them under a generic metamodel concept. We explain how different AC components can be defined, instantiated to derive various models from the HEAD metamodel, and illustrate several scenarios to show its dynamism and extensibility. Additionally, we show how our metamodel supports the feature of hierarchy for all components, which is essential to define policies in, for example, a hierarchical organizational structure where, for example, several users might be assigned to several roles in a hierarchy and have permission to access several objects also in a hierarchy. However, the contribution of this paper can be summarized as follows:

- Proposing an advanced AC metamodel with generic, dynamic, and extensible characteristics; it can also be considered a foundation stone to solve other limitations in existing AC metamodels, including collaboration and interoperability between various models.
- Providing a solution for the need of component hierarchy in AC models (e.g., objects, actions, roles, categories, contexts, etc.).
- Developing a language to express AC requirements, and to serve as a basis for producing AC decisions for access requests.
- Providing a grammar language that is simple and flexible to appropriately express AC policy requirements.
- Assisting developers and security experts to include unified and generic components in designing secure ISs that conform to the organizational AC security policies.

The remainder of this paper is organized as follows. In Section 2, we review the related works in this domain. In Section 3, we explain how we unify the common concepts of heterogeneous AC models, then we present the HEAD metamodel with its kernel elements based on the unified concepts. In Section 4, we design the tools for metamodel instantiation. We use the Eclipse Xtext framework to define the domain-specific language (DSL) of our metamodel, and then we explain the metamodel characteristics. In Section 5, we show how the HEAD metamodel provides a generic base to include all components of AC models and is able to derive various models (also hybrid models) instances. We also represent several scenarios to show its dynamicity, extensibility, and how it supports the hierarchy of different components. In Section 6, we illustrate some examples of how AC policies can be generated, using the HEAD metamodel. Section 7 concludes this paper with future perspectives.

## 2. Related Works

As mentioned earlier, different AC implementations to define and enforce AC policies have been presented in the literature over the decades. In this section, we summarize the proposed AC models, starting from traditional AC models reaching to the motif of AC metamodels.

The traditional AC models are DAC, MAC, RBAC, and ABAC [6]. In DAC, mapping users to operations on an object often takes the form of an access control matrix, while MAC is based on the concept of security levels assigned to subjects and objects to control the direction of information flow and users’ operations. In these models, AC decisions are based on the identity of the user, and they are inadequate in dynamic computing environments where the sharing of information between systems and users from diverse security domains is common [5,8]. Thereafter, the RBAC model is proposed to provide a more generalized model than DAC and MAC. In RBAC, if users are assigned to a role, then they are granted a set of permissions. This model is insufficiently flexible for several scenarios and makes administration potentially heavy in very large systems [11,15], for example, systems with several local branches and abroad (e.g., banks), where hundreds of users need to be assigned to roles to determine their permissions. Additionally, it does not support dynamic attributes (e.g., time of day), for example, to prevent users from accessing any information after their working hours. Furthermore, due to continuous technology developments, the demand for a more generic and dynamic AC model has grown, and thus, the ABAC model is proposed. In ABAC, AC decisions are based on the attributes of users (subjects), attributes of objects being accessed, and attributes of environment (context). One of the ABAC model’s limitations is the difficulty to calculate the resulting set of permissions for a given user [5,6,15,16].

Due to continuous technology growth and the appearance of distributed systems and because of some shortfalls in each of the traditional models [10,17], different approaches are proposed in the literature to enhance AC methods by combining features of two or more AC models, called hybrid models. A hybrid model is proposed by Kaiwen and Lihua in [18], based on attribute and role, to solve the shortage on the environment of large-scale dynamic users by solving the aspect of permissions assignment and policy management. Another hybrid RBAC/ABAC model approach is presented in [19] for multi-domain information systems. To find a model that supports dynamic attributes and handle relationship between roles and attributes to provide better AC features in dynamic environments, Kuhn et al. in [20] address the idea of adding attributes to RBAC. Moreover, Rajpoot et al. [7] integrate RBAC and ABAC models by associating attributes with subjects, objects and the environment, allowing the request context to be considered in making AC decisions. In [21], a hybrid model merging RBAC and ABAC is proposed to enhance the dynamic features of RBAC and to provide ease of administration, tight security, dynamic behavior, and efficient separation of duty implementation. Additionally, Kim et al. in [22] propose the MAC/RBAC model by configuring RBAC and MAC features to be applied in the domains where access has to be checked for both authorized roles and security levels together, for example, in the hospital domain, government domain, and military domain.

Moreover, to enhance an AC model in order to define additional rules, some AC models are extended by adding new component(s) to them. For example, in [23], a survey of an extended RBAC model in cloud computing is presented. In addition, an ABAC model extension is presented in [8], called hierarchical group and attribute-based access control (HGABAC), where groups and hierarchies of subjects and objects are added. Another ABAC extension is proposed in [9], named the higher-order attribute-based access control model (HoBAC); it extends the basic concepts (subjects, objects, and contexts) of the model with aggregation operations that provide hierarchies, and many other examples. As well, in [24], the RBAC model is abstracted to the meta-level in order to support the delegation of users’ rights, which allows a user without any specific administrative privileges to delegate their access rights to another user; hence, a delegation metamodel is proposed for specifying RBAC and RBAC-based delegation features. In [25], Adda and Aliane proposed a generic ABAC metamodel to generate a wide variety of AC models related to the ABAC model that is suitable for all computing environments.

The current generation of networking environments impose the need to focus on developing more advanced AC features, especially since the existing AC models—hybrid models, extended AC models, and abstract AC models—have reached their limits and are currently insufficient to meet the needed AC requirements [5,17]. What makes this fact challenging is the heterogeneity of everything—networks, applications, devices, etc.—in addition to the heterogeneity of AC models. Hence, the notion of AC metamodels has existed for almost a decade [11]. They are proposed to work as frameworks to allow instantiating various models, and allow defining and enforcing a larger set of AC policies. These metamodels are proposed to include most of the features and components of AC models, which are employed in the core metamodel structure [5,11]. Hence, the more features they include, the more they allow defining a larger set of AC rules.

In the literature, several metamodel approaches are proposed for different computing environments. A unified AC modeling language is proposed in [26] as an extension for Barker’s metamodel [27] to support object and action hierarchies. The aim of their language is to define hybrid AC policies by allowing categories to be associated with other categories and finding hierarchical associations between them. A subject may be assigned to a category, which could be a role, a class, a group, a security level, etc. Barker’s approach includes features of MAC, DAC, and RBAC, and is named the category-based access control metamodel (CBAC). Another approach is proposed by Bertolissi et al. in [28] for distributed environments and is based on the CBAC metamodel, where the user’s request can be passed to other sites and evaluated in a distributed manner. An integration metamodel for hybrid policies to concurrently handle DAC, MAC, and RBAC models is proposed by Abd-Ali et al. in [29]. Alves et al. in [30] propose a CBAC metamodel extension to study the interaction between obligations and permissions and expand a general notion of obligation for the existing AC models. A category-based access control (CatBAC) metamodel for highly flexible and dynamic environments, specifically for cloud computing services, is proposed by Khamadja et al. in [31], to allow security administrators in various company sites to find a concrete model with the constraints and specificities of each site. To handle security and privacy in cloud service development and operations, Xia et al. in [32] propose the cloud security and privacy metamodel (CSPM). To facilitate the analysis and manipulation of security requirements for web services for the representation of web content management systems (WCMS) AC policies and to automatically extract the AC information in the domain of WCMS, a metamodel approach is presented by Martínez et al. [33].

Generally, the metamodels are proposed to address the notions of including various hybrid AC models features in the core metamodel structure, to encompass AC models and allow extending them by adding new components, and to find a generic structure that could include the most possible features of AC models. Nevertheless, the proposed AC metamodels have several limitations and lack some essential characteristics [5,11,34], and can be summarized as follows:

- They do not include all features of the common AC models, so they are not generic enough;
- They do not support the ability of defining new entities and building relationships between them in order to describe larger set of AC rules, for example, due to changing environmental conditions, so they are not dynamic enough to follow technology upgrades;
- They do not include the possibility of defining new components and attributes in addition to the defined ones, so they are not extensible and the defined policies cannot be extended;
- They do not support the feature of hierarchy for components;
- They do not handle or support the feature of collaboration and interoperability between the various AC models;
- They do not tackle the issue of migrating AC policies from one model to another.

In this paper, our concern is to provide solutions for the limitations of generality, dynamism, extensibility, and hierarchy.

## 3. Formalization of Access Control Policies

A security policy is a definition of a set of rules and guidelines on which access is granted or denied for a user in any organization or industry sector. The following are some general examples of AC rules:

- Before check-in, each worker has to wear a face mask.
- The maximum number of visitors in each room is 15.
- Machine operators can only enter the labs during working hours.

To define a policy, a set of concepts are defined and formulated to form rules. For example, in the above rules we have subjects (e.g., worker, operator), objects (e.g., face-mask, machine, room), actions (e.g., wear, visit, enter), attributes (e.g., maximum number, working hours), and many other concepts could be included in a defined policy, such as permission, role, group, etc.

### 3.1. Unifying Access Control Concepts of Heterogeneous Security Policies

In the literature, several types of AC models are implemented; these models can be defined as frameworks for making authorization decisions. Each model is formulated based on AC concepts. The following are some examples:

- The DAC model includes subject, object, and action concepts.
- The MAC model includes subjects, object, security level, and operation concepts.
- The RBAC model includes subject, object, role, permission, and action concepts.
- The ABAC model includes subject attributes, object attributes, context attributes, condition, and action concepts.

Consequently, we can find that security policies include common concepts and attributes that are common to all AC models [10]. These concepts can be summarized as follows:

1. A set of concepts (and attributes) to describe subjects and objects.
2. A set of concepts (and attributes) that describe the authorized subjects.
3. A set of concepts (and attributes) that explain the different access rights.
4. A set of concepts (and attributes) that set various constraints and conditions.
5. A set of concepts (and attributes) that describe the context (environmental context) to access objects.

**Figure 2:**
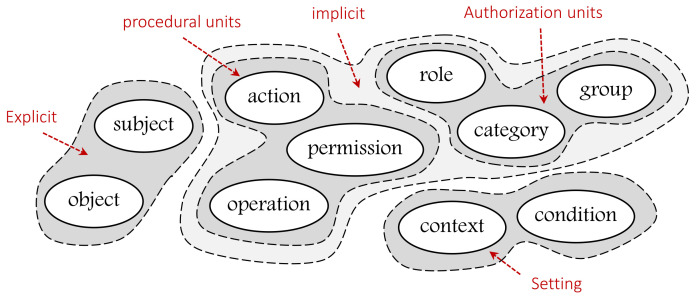
Unifying heterogeneous concepts of AC models.

To unify them and make them adaptable to all AC models, we classify them into explicit, implicit (authorization units and procedural units), and setting concepts as illustrated in Figure 2. Note that each of the classified groups may include additional concepts. EXPLICIT concepts are those that refer to something that is real and exists (e.g., subjects and objects). IMPLICIT concepts are those that refer to something described or explained in the guidelines or rules. Implicit concepts include AUTHORIZATION UNITs (e.g., roles, security levels…) and PROCEDURAL UNITs (actions, permissions…). SETTING refers to concepts that are included to have more accurate and regulated access to resources (e.g., context, constraints…).

### 3.2. HEAD Metamodel

The key responsibility of an AC metamodel is to define a language for specifying several AC models; usually, this level describes generic concepts. Examples of meta-objects (or meta-concepts) at the meta-modeling layer are the following: class/entity, attribute, component, and others. An AC model is an instance of the metamodel. The key responsibility of the model is to define a language that describes a security policy. Examples of objects (or concepts) at modeling layer are subjects, objects (or resources), actions, and other concepts; this level explains how these objects work together. At the system layer where users interact, the actual AC policies are expressed by a security expert for a given AC model instance(s). Note that in this section, we use the term “component” instead of “concept”. Table 1 gives a summary about the meta-modeling layers and details of our approach.

**Figure 3:**
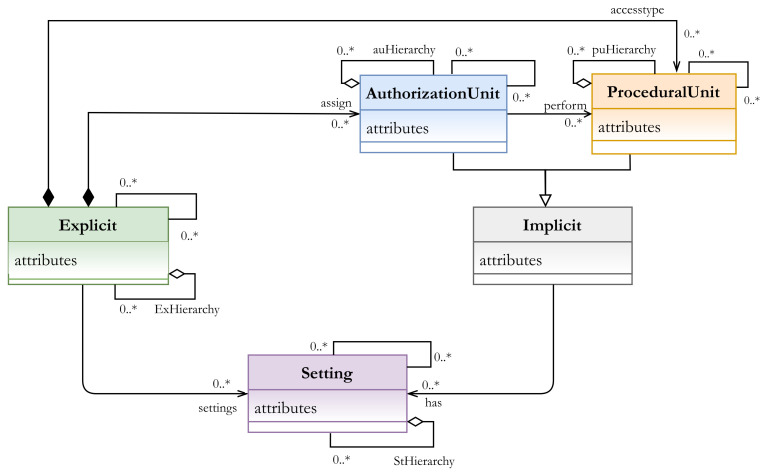
HEAD metamodel: the kernel elements.

Our AC metamodel aims first to describe the AC policy at the abstract level that is autonomous from the enforcement of this policy. Accordingly, instead of modeling the AC policy by using the concrete components of subject, object, permission, action, etc., we define the meta-policy by using the abstract components of explicit, implicit, and setting, then instantiate the concrete components to model the needed policy. The main characteristics of the HEAD metamodel are as follows (Figure 3):

- It unifies the heterogeneous components of AC models.
- It is generic enough to include the common AC models and other models.
- It is dynamic and includes the feature of defining components (and attributes for all components).
- It is extensible since it allows extending the already derived AC models.
- It supports the hierarchy for any type of components.

Unifying the heterogeneous components of AC models and grouping them based on their functionality would allow instantiating an unlimited number of components related to the meta-component. Another essential characteristic is its generic structure, where all components of common AC models can be defined and new ones can also be defined. In other words, it is not restricted to the common models and it can also be used as a base to derive new models. Additionally, unlimited levels of hierarchy could be defined for all components whether they belong to explicit, implicit, or setting concepts. An AC metamodel that supports the hierarchy of components is an essential characteristic and cannot be ignored with the current distributed and complex structures of computing environments and the existing resources.

#### 3.2.1. Kernel Elements: HEAD Metamodel

In this section, we present the kernel elements (meta-components) of our metamodel and the relationships between them.

- EXPLICIT (E_x_): a set of explicit components that represent the real and the existing entities, such as subjects and objects in any organization or industry sector. The class EXPLICIT has a composition association with the sub-classes AUTHORIZATION UNIT and PROCEDURAL UNIT, which are the inheritance of the abstract IMPLICIT class.
- IMPLICIT (I_m_): a set of implicit components that represent the described components. For example, subjects are classified or assigned to some other component(s) (e.g., roles), or the processes or functions that can be performed (e.g., actions). Two other sub-classes that are inherited from the Implicit super-class are the AUTHORIZATION UNIT (AU) components and the PROCEDURAL UNIT (PU) components.
  - AUTHORIZATION UNIT (AU): a set of authorization units. It is a subclass which should be specialized to create specific authorization units ($au_{i}$, sub-index i specifies the unit type), such as roles, categories, security levels, etc., to which some E_x_ units (e.g., subjects) can be assigned, An AU example is as follows:
    - au${}_{i}$: role (manager, doctor, …)
    - au${}_{j}$: category (age > 18, temperature < 38${}^{\circ }$, …)
    Hence, AU includes role and category components
  - PROCEDURAL UNIT (PU): a set of procedural units. It is a subclass which should be specialized to create specific procedural units ($pu_{i}$, sub-index i specifies the unit type) such as actions, permissions, operations, etc., to which some E_x_ units (e.g., objects) can be assigned. In other words, it represents operations that can be performed by E_x_ units (e.g., subjects) on some other E_x_ units (e.g., objects), a PU example is as follows:
    - pu${}_{i}$: action (read, write, …)
    - pu${}_{j}$: operation (turn on/off, open, close, …)
    Hence, PU includes action and operation components.
- SETTING (S_t_): a set of setting components. It represents the concepts that are included to have more accurate and regulated access to resources, for example, context, contextual conditions, constraints, etc. The setting components actually provide our metamodel with high flexibility and expressiveness. They could include other components (explicit, implicit, or/and other setting) to construct the needed expression(s). For example, A context expression and contextual conditions can be expressed in terms of AU, PU, E_x_, and S_t_ components.

#### 3.2.2. Hierarchies and Associations

**Figure 4:**
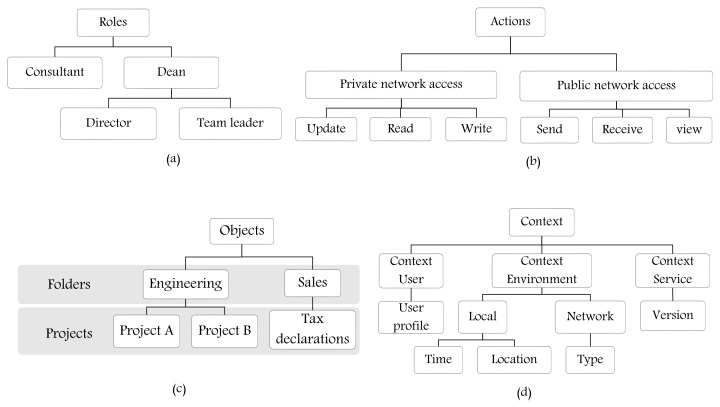
Examples for hierarchy of (**a**) roles; (**b**) actions; (**c**) objects; and (**d**) contexts.

The concept of hierarchy is important to define multiple levels of components, such as roles, actions, objects, etc. It reflects the structure of an organization and, for example, the respective responsibilities/priorities of the hierarchical components. Figure 4 represents some examples of hierarchy in an organization or industry sector. In our metamodel, there are four basic sets of components: E_x_ (set of explicit entities/classes), AU (set of authorization unit entities/classes), PU (set of procedural unit entities/classes), and S_t_ (set of setting entities/classes). Our metamodel provides support for creating hierarchy for classes of AU (e.g., role hierarchy Figure 4a), PU (e.g., action hierarchy Figure 4b), E_x_ (e.g., object or resource hierarchy, Figure 4c), and S_t_ (e.g., context hierarchy, Figure 4d) by aggregating AU, PU, E_x_, and S_t_ entities. These hierarchical relationships are depicted by an aggregation association in Figure 3.

The association between E_x_ and AU is to assign, for example, zero or many (0..*) subjects to roles, groups, categories or other AUs. The association between AU and PU and PU and E_x_ is to represent which AUs are able to perform zero or many PUs (e.g., actions, permissions …) and access some, for example, objects or services. Note that I_m_ components (AUs and PUs) might have zero or many S_t_ (e.g., contextual and/or non-contextual constraints) before accessing/performing tasks on E_x_ components. Moreover, the metamodel provides support for formulating AC models and hybrid models for different policies by allowing AUs to be associated with other AUs, PUs to be associated with other PUs, E_x_ components to be associated with other E_x_ components, and S_t_ components to be associated with other S_t_ components. As shown in Figure 3 a self-association edge exists on each of the classes. Note that in some models, we might have an empty set of AU, or S_t_, for example, in the DAC model, AU is an empty set since explicit components are not assigned to AUs.

#### 3.2.3. Meta-Policy and Policy

In this section, we explain the notions of meta-policy and policy of the HEAD metamodel; note that the theoretical foundations of the HEAD metamodel are not included in this paper. The meta-policy is expressed using the meta-components of E_x_, I_m_, and S_t_, in the following way:

$$Metapolicy=\langle E_{x},I_{m},S_{t}\rangle$$

Based on this meta-policy, different AC policy definitions can be expressed as follows:

- To define RBAC policy:
  - E_x_ = {subject,object}
  - I_m_ = {AU=role,PU=permission}
    Hence, Policy=〈subject,object,role,permission,action〉
    Meaning that a subject assigned to role has permission(s) to access object(s) and perform action(s).
- To define a hybrid MAC/RBAC policy:
  - E_x_ = {subject,object}
  - I_m_ = {AU=role,securitylevel,PU=permission,action}
    Hence, Policy=〈subject,object,role,securitylevel,permissions,actions〉
    Meaning that, subjects who are assigned to specific roles and security levels have permissions to access objects that are classified to some security levels and perform some actions.
  Thus, the policy is expressed using model components, which are derived from the meta-components of meta-policy.

An AC policy is a set of rules that determine users’ access rights within a given information system. These rules constitute a definition of the AC requirements for the system. The process of implementing the AC mechanisms to make the system follow the defined rules is called enforcement. In this paper, our concern is to constitute the definition of AC requirements for a system.

## 4. Defining the Grammar of HEAD Metamodel

In the literature, several AC models, such as MAC, DAC, RBAC, ABAC, and many other hybrid models are formulated based on the definition of security rules. Depending on the model, the type of rules and the components (or entities) they employ are different. The remarkable advantage of our metamodel is that it supports the definition of AC policies for all these models and allows the implementation of generic tools to derive them. To handle this idea, the metamodel must allow defining the different components and attributes, then expressing models using them.

**Figure 5:**
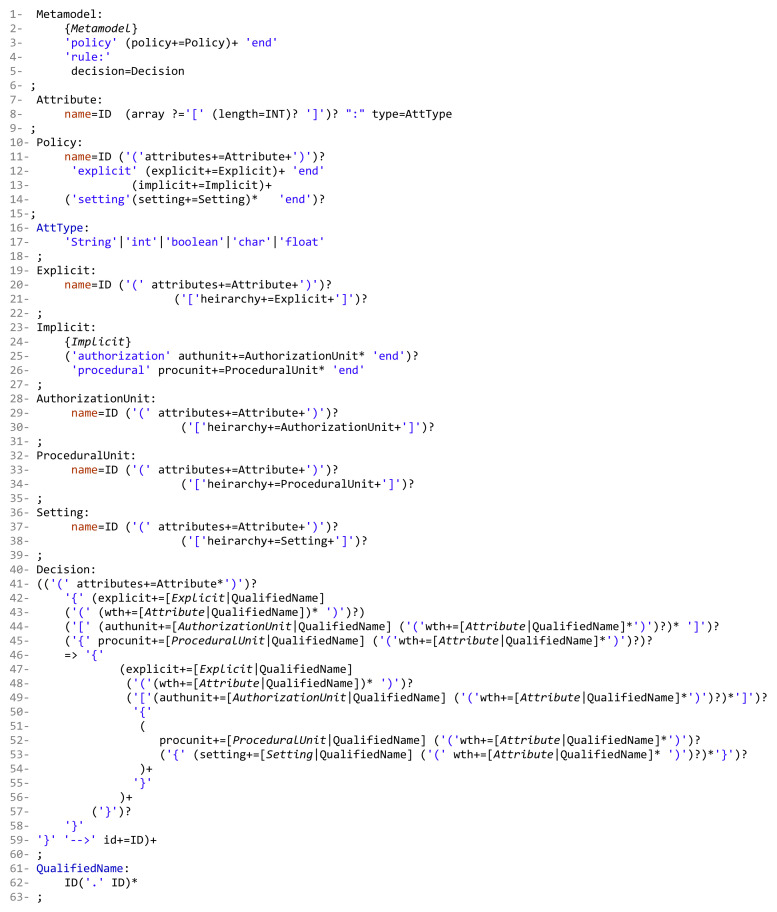
HEAD Metamodel: The Grammar.

This section addresses the definition of the grammar of the DSL for our AC metamodel; the grammar we have created is listed in Figure 5. Our grammar definition can be interpreted as follows:

1. **Lines 1 to 39**: to instantiate the needed AC model(s) components, the hierarchies, and the attributes.
  - Lines 1 to 6: the block of defining all model components. ‘Metamodel’ is the root class for the definition of parser rules. The used keywords ‘policy’ and ‘end’, in line 3, are used to indicate the start and end of creating policy components. Note that our metamodel is able to create one or more policy types (e.g., MAC policy and RBAC policy). Each defined rule generates one decision (line 5).
  - Lines 7 to 9: the declaration of attribute(s) name(s) and datatype(s); also, arrays can be declared.
  - Lines 10 to 15: the definition of the policy name (e.g., RBAC) and the sub-blocks (inside the main block of policy) of the E_x_, I_m_, and S_t_ components. To create a policy, at least one or more explicit/implicit element(s) must be declared; also, we could have zero or more setting element(s). To define the sub-block of E_x_ and S_t_ elements, the keywords ‘explicit’ and ‘setting’ are used, respectively, at the beginning, and ‘end’ at the end. Note that the ‘Implicit’ parser rule (line 13) has two elements, ‘AuthorizationUnit’ and ‘ProceduralUnit’ (lines 23 to 27).
  - Lines 16 to 18: the alternatives of attribute data types.
  - Lines 19 to 22: the creation of E_x_ components, their attributes, and their hierarchies.
  - Lines 23 to 27: the creation of sub-blocks of I_m_ elements (AUs and PUs). The keywords ‘authorization’ and ‘procedural’ are used to indicate the beginning of each sub-block, and ‘end’ at the ending.
  - Lines 28 to 31: the creation of AU components, their attributes, and hierarchies.
  - Lines 32 to 35: the creation of PU components, their attributes, and hierarchies.
  - Lines 36 to 39: the creation of S_t_ components, their attributes, and hierarchies.
  - Note that attributes could be defined for all components, and an unlimited number of levels for components hierarchy can be created.
2. **Lines 40 to 63**: to define a policy (set of rules), based on the instantiated components and attributes with the access request decision. Note that, using our grammar definition, rules (and hybrid rules) can be expressed in different ways, for example, a subject can access object(s) and perform an operation(s), or an object can be accessed by a subject(s) and perform an action(s).
  - Line 40: the parser rule ‘Decision’, is the beginning of specifying and expressing a rule which ends with a decision (‘–>’ id+=ID, line 59).
  - Line 41: after using the keyword ‘rule’ (line 4), some attributes can be created, for example, ruletype, rulenumber, etc.
  - Lines 42 and 44: the block of rule definition starts with an open curly braces ‘{’. A rule is started by specifying an E_x_ component (e.g., subject or object) and its attributes. In some models, explicit components are assigned to some AUs, for example, in RBAC subjects are assigned to roles, and in MAC subjects/objects are assigned to security levels. Note that E_x_-AU assignment is optional ‘?’ in expressing a rule, depending on the expressed model (line 44).
  - Line 45: it is optional to define a nested block for a procedural unit, for example permission in RBAC, to express a policy.
  - Line 46: the beginning of expressing another nested block after specifying an E_x_ component and assigning it to some AU(s), or defining some PU(s).
  - Lines 47 to 49: same interpretation of lines 42 to 44. Hence, the beginning of a rule could be expressed as follows, for example:
    - A subject can access object(s)…
    - An object can be accessed by a subject(s)…
    - A subject assigned to a role has permission to access object(s)…
    - A subject assigned to a security level can access object(s) assigned to some security levels…
  - Lines 47 to 56: the sign ‘+’ in ‘)+’, line 56, indicates that what is included between lines 47 to 56 can be expressed more than once within a rule.
  - Lines 50 to 55: the start and end of a sub-block of specifying what PUs (e.g., actions) an E_x_ unit can perform. Note that it is optional to include some S_t_ (e.g., conditions) while expressing a rule. The sign ‘+’ in ‘)+’, line 54, indicates that what is included between lines 51 and 54 can be expressed more than once.
  - Line 57 to 59: closing the main block with some of opened sub-blocks.
  - Line 59: indicates the end of rule expression with the decision ID. Note that the sign ‘+’ in ‘)+’indicates that a set of rules can be defined within a policy.

In Section 5, we explain with examples how our metamodel grammar could be expressed to define different rules, and show how it is generic, dynamic, extensible, and supports a hierarchy of components.

## 5. Deriving Access Control Models

In this section, we show how our metamodel structure is (1) generic and able to derive instances of different models and hybrid models, (2) dynamic and allows defining new components in addition to the existing ones, also the relationships between them, (3) extensible to upgrade any defined policy, and (4) supportive of the feature of defining hierarchies.

The key responsibility of the model is to define a language that describes a security policy. Examples of entities at modeling layer are: subjects, objects (or resources), actions, and other entities. This metamodeling layer explains the way of how these entities work togather. Henceforth, we use the term “entity” instead of “component”. Note that in this paper, we consider the environmental context.

### 5.1. Generality

**Figure 6:**
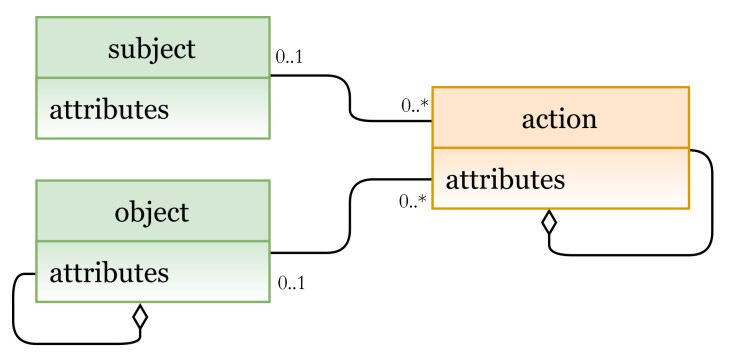
DAC model instance.

In this section, common models (DAC, MAC, RBAC, and ABAC), in addition to some hybrid models, are instantiated, using the HEAD metamodel, and show how different AC rules can be expressed using the defined grammar (Figure 5). The models in Figures 6, 8, 10, 12, 14 and 16 illustrate class instances with the same colors of EXPLICIT, IMPLICIT, and SETTING classes of Figure 3.

#### 5.1.1. Discretionary Access Control Model (DAC)

In DAC, subjects determine how some other subjects can access their objects [35]. It is based on the identity of three key entities shown in Figure 6, the E_x_ entities are subject and object, and the I_m_ entity is operation (PU instance). Subjects can control access rights to their objects by determining what operations can be performed by other subjects.

**Figure 7:**
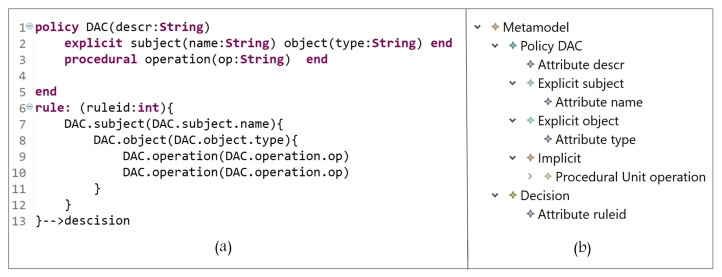
(**a**) DAC Policy Definition; (**b**) DAC entities.

Based on our grammar definition, in Figure 7a (lines 1 to 4), we define DAC entities (and the needed attributes), and their instances are shown in Figure 7b. The DAC policy is expressed in lines 6 to 13 starting with the keyword ‘rule’, and since a policy is a set of rules, we define the attribute ‘ruleid’ to indicate the rule number. Note that any rule could have allow, deny, mixed, etc., decisions. Hence, the rule can be interpreted as follows:

A subject with name = name-value can access object with type = type-value and perform some operation(s) op = op-value(s).

#### 5.1.2. Mandatory Access Control Model (MAC)

**Figure 8:**
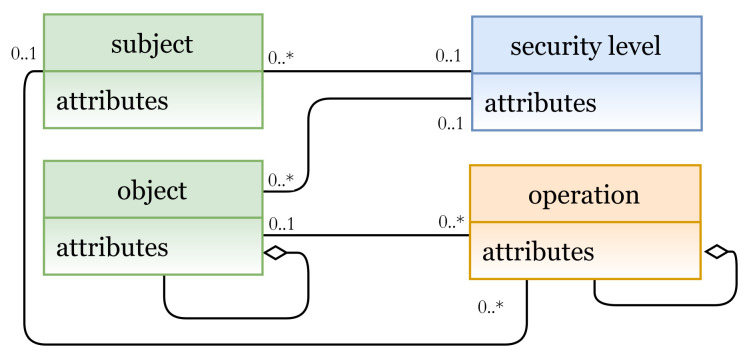
MAC model instance.

In MAC, access rights are based on the concept of security levels associated with each subject and object, where actions are derived. A security level for a subject is called the clearance level and for an object is called the classification level [6]. In Figure 8, the E_x_ entities are subject and object, and the I_m_ entities are security level (AU instance), and operation (PU instance). Clearance levels are assigned to subjects and objects, and based on these levels, AC rights are specified.

**Figure 9:**
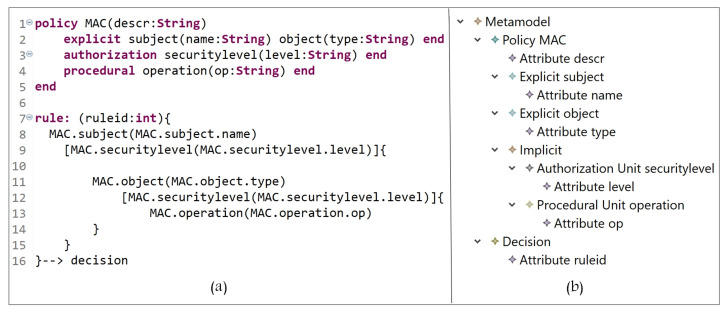
(**a**) MAC policy definition; (**b**) MAC entities.

In Figure 9a (lines 1 to 5), we define MAC entities (and the needed attributes), and their instances are shown in Figure 9b. The MAC policy is expressed in lines 7 to 16, starting with the keyword ‘rule’, and the attribute ‘ruleid’ to indicate the rule number. Note that any rule could also have allow, deny, mixed, etc., decisions. Hence, the rule can be interpreted as follows:

A subject with name = name-value which is assigned to a securitylevel = clearancelevel-value can access object with securitylevel = classificationlevel-value and perform some operation(s) op = op-value(s).

In MAC, for example, the BLP (Bell–LaPadula) model, a subject is allowed to read an object if its clearance level is greater than or equal to the object’s classification level [6].

#### 5.1.3. Role-Based Access Control Model (RBAC)

**Figure 10:**
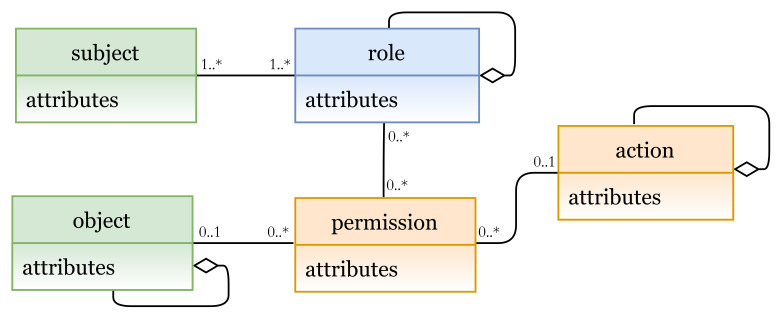
RBAC model instance.

In RBAC, subjects are given access based on their roles (e.g., engineer and doctor). In Figure 10, the E_x_ entities are subject and object, and the I_m_ entities are role (AU instance), and permission and action (PU instances). Subjects can be assigned to different roles (roles can be associated to several subjects), and each role is a group of permissions to perform some actions. As mentioned earlier, our metamodel provides support for creating hierarchies by aggregating some concepts. As shown in the figure, hierarchies for objects (resources), roles, and actions can be created [35].

**Figure 11:**
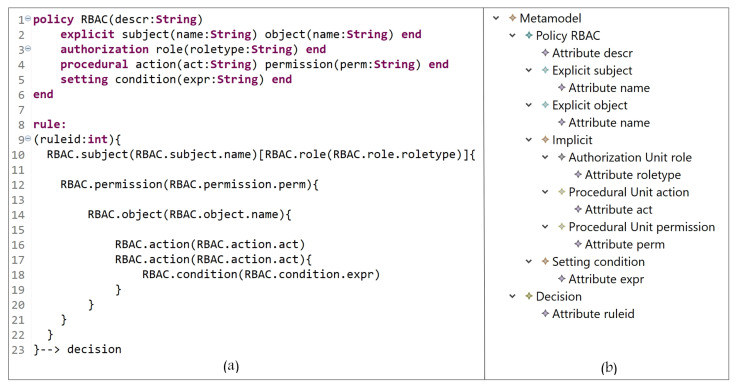
(**a**) RBAC policy definition; (**b**) RBAC entities.

In Figure 11a (lines 1 to 6), RBAC entities (and the needed attributes) are defined, using our defined metamodel language; their instances are shown in Figure 11b. RBAC policy is expressed in lines 8 to 23. RBAC rule can be interpreted as follows:

A subject with name = subjectname-value assigned to a role roletype = role-value has the permission to access object name = objectname-value and perform action act = act-value1, and action act = act-value2 if condition expr = condition-expression is true.

Note that the ‘act’ attribute might have read value for the first action and write for the second action.

#### 5.1.4. Attribute-Based Access Control Model (ABAC)

**Figure 12:**
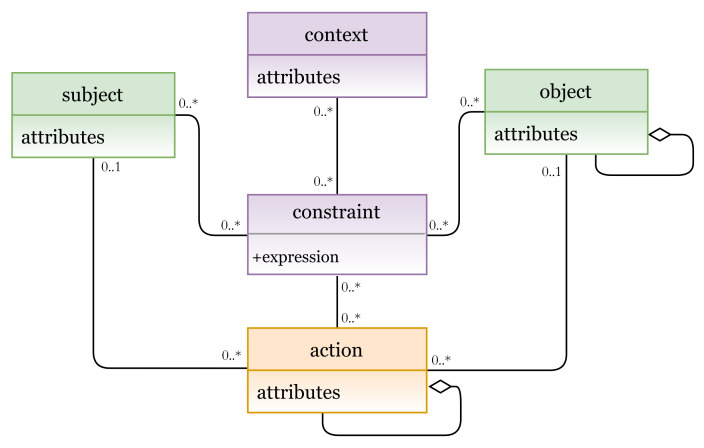
ABAC model instance.

In the ABAC model, AC rights are evaluated at the time that the actual request is made; it uses subject, object, and environmental (context) attributes to determine access decisions. In Figure 12, the E_x_ entities are the subject attributes and object attributes (which represent subjects and objects); the I_m_ entities are permission and action (PU instances); the S_t_ entities are context expressions and attributes. Subjects with some attributes are allowed to perform some actions on objects with some other attributes based on some conditions and constraints in the defined policy.

**Figure 13:**
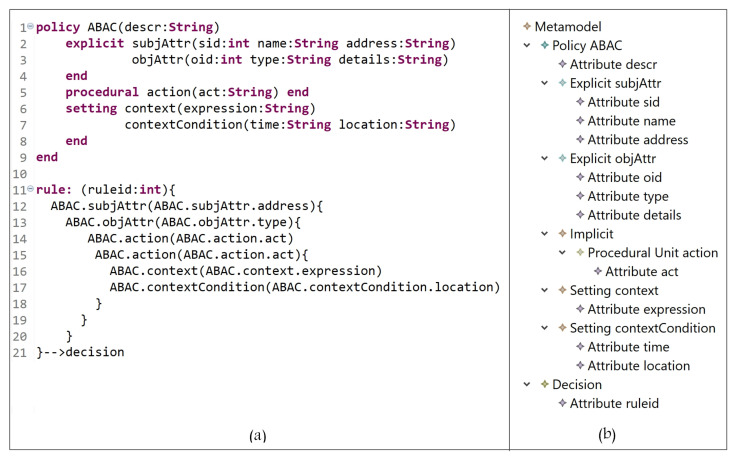
(**a**) ABAC policy definition; (**b**) ABAC entities.

In Figure 13a (lines 1 to 9), ABAC attributes, which represent subjects, objects, actions, and context, are defined; the instances are shown in Figure 13b. ABAC policy is expressed in lines 11 to 21. A rule can be interpreted as follows:

A subject with address = address value, and … attributes can access object with type = type value, and … attributes and perform an action act = act-value1, and act = act-value2 when expression if location = location-value is true.

Note that the ‘act’ attribute might have update for the first action and delete for the second action.

#### 5.1.5. Hybrid Models

**Figure 14:**
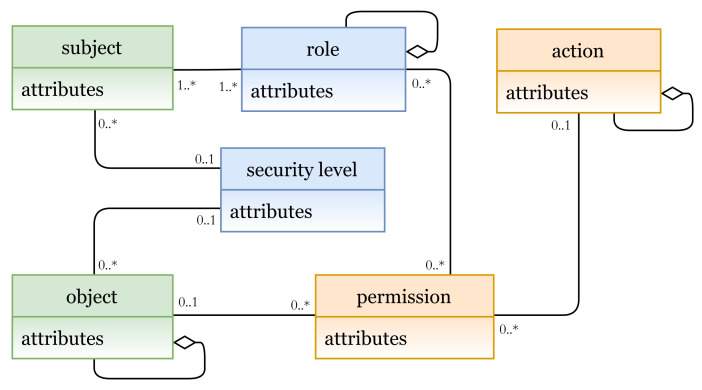
Hybrid MAC/RBAC model.

A hybrid AC model combines features of two or more AC models. Using the grammar of the HEAD metamodel, various hybrid models can also be instantiated. Figure 14 represents a hybrid MAC/RBAC model instance.

**Figure 15:**
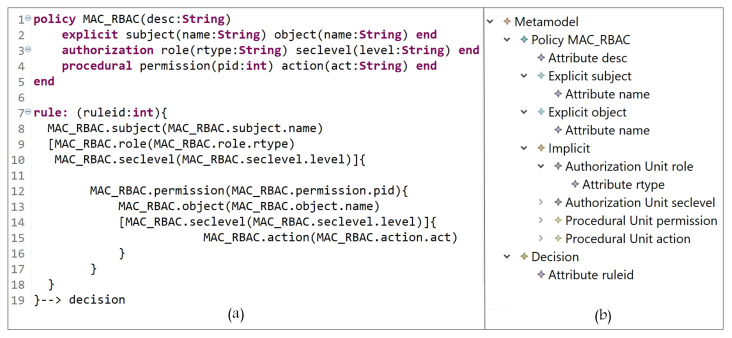
(**a**) MAC/RBAC policy definition; (**b**) MAC/RBAC entities.

In Figure 15a (lines 1 to 5), we define MAC/RBAC entities (and the needed attributes), and their instances are shown in Figure 15b (subject, object, security level, role, action, and permission). A hybrid MAC/RBAC policy is expressed in lines 7 to 19, with the attribute ‘ruleid’ to indicate rule number. A MAC/RBAC rule can be interpreted as follows:

A subject which is assigned to role = roletype-value and securitylevel = clearancelevel-value has a permission to access object(s) with securitylevel = classificationlevel-value and perform some action(s) act = act-value.

**Figure 16:**
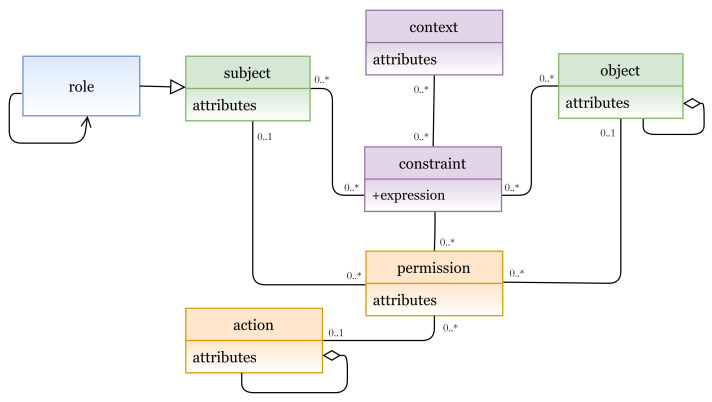
Hybrid RBAC/ABAC model.

Another hybrid model example is illustrated in Figure 16, which represents an instance of the hybrid RBAC/ABCA model. In this hybrid model, to determine subject’s role, a role is added as an attribute to the subject entity.

**Figure 17:**
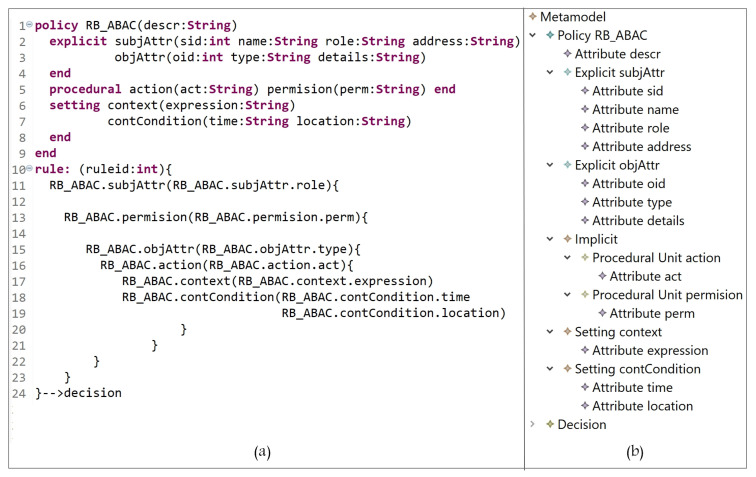
(**a**) RBAC/ABAC policy definition; (**b**) RBAC/ABAC entities.

In Figure 17a (lines 1 to 9), we define the RBAC/ABAC entities and attributes, and their instances are shown in Figure 17b (subject attributes, object attributes, action, permission, context, and contextual attributes). A hybrid RBAC/ABAC policy is expressed in lines 10 to 24. A rule can be interpreted as follows:

A subject with attributes address = address-value and role = role-value has a permission to access an object(s) with attribute(s) type = type-value and perform an action where act = act-value when context-expression if location = location-value and time = time-value are true.

As shown above, various AC models can be instantiated using the HEAD metamodel; hence, it is generic and able to include any AC feature for any model, and is also flexible enough to define the needed AC policies (also hybrid policies).

### 5.2. Dynamism

Along with technology upgrades, several security threats appear. To conquer them, security solutions must be regularly updated and stay amenable to follow and track the evolution of these threats. Protecting resources against security threats has become a crucial concern in the development of IS and requires setting up trusted AC policies. The HEAD metamodel exceeds the features of the existing metamodels since it considers that AC is becoming more and more important for open, ubiquitous, and critical systems. An AC metamodel must be flexible and upgradable, due to changing conditions or updating rules. In other words, its structure should be dynamic and describe how its properties can be adjusted over time to define a larger set of static and dynamic AC policies. Hence, a dynamic metamodel allows defining new types of attributes, for example, contextual attributes, and new components, in addition to the relationships between them to upgrade and formulate different AC models. In this section, we assume some scenarios as examples to show the dynamism of the HEAD metamodel.

**Figure 18:**
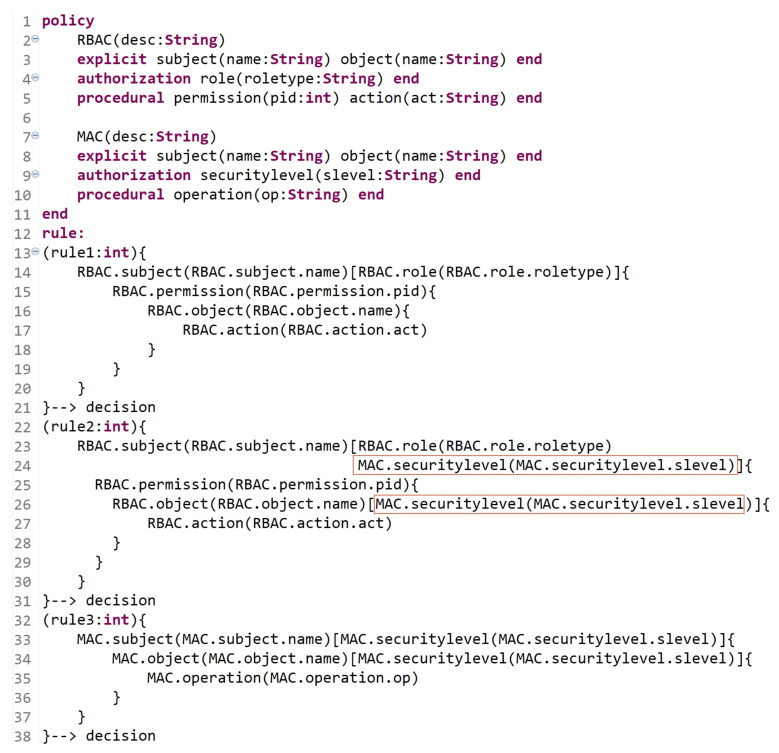
Dynamic AC metamodel: Scenario 1.

**Figure 19:**
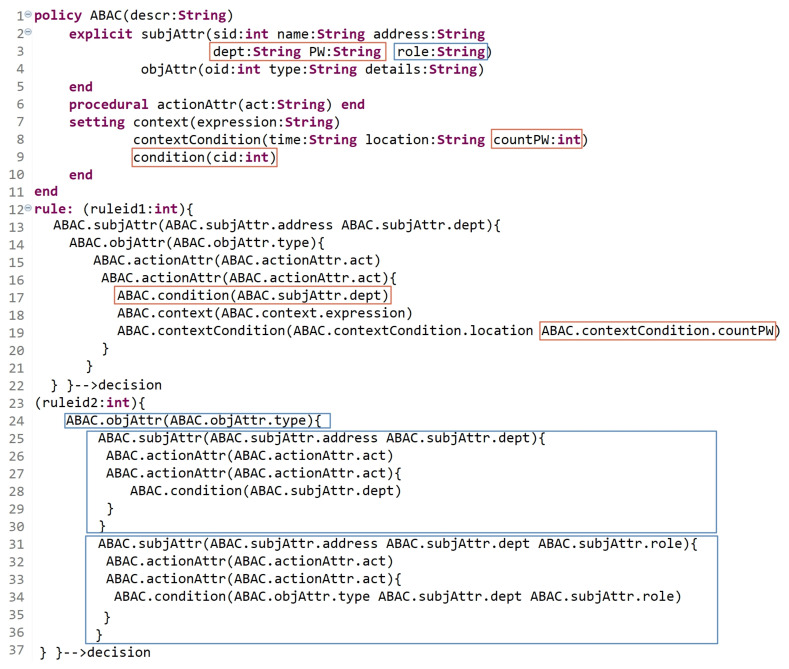
Dynamic AC metamodel: Scenario 2.

- Scenario 1: Assume that an RBAC model is already formulated to define a policy for an organization. Hereinafter, due to some organizational changes and updates, the following occurrs:
  - Some users who are already assigned to certain roles need to be assigned, based on their roles, to security levels.
  - Some other users now should be directly assigned to levels.
  - Both users in (a) and (b) are only allowed to access some sensitive objects (e.g., documents), which are also classified into levels based on their sensitivity.
  Hence, some of already defined rules must be updated, and new rules must be defined. Figure 18 is an example of how the HEAD grammar is dynamic. Lines 2 to 5 indicate the already defined RBAC entities (with the attributes), and lines 13 to 21 indicate the already defined RBAC rules. Lines 22 to 31 indicate that some RBAC rules are updated to express hybrid MAC/RBAC rules. As we can see, in line 24, some subjects who are assigned to some roles are now assigned to some security levels, and in line 26, some objects are assigned to some security levels. Lines 32 to 38 indicate the definition of new MAC rules.
- Scenario 2: Assume that an ABAC model is already formulated, and the policy is already defined in an organization. Suppose that the organization has departments dept1, dept2, and dept3. However, due to some changing conditions, a new static and dynamic AC rules must be defined and others must be updated. The updated policy, due to new changing conditions, states the following:
  - Dynamic rule: subjects (users) in dept2 and dept3 are not allowed to access some objects after three failed password attempts (assuming that another level of authentication is needed before accessing the objects).
  - Static rule: some subjects in dept1 can determine what operations, (i) other subjects can perform, and (ii) some other subjects with the specific role can perform, on their objects.
  Clearly, some already defined rules must also be updated, and new rules must be defined. As shown in Figure 19, the red and blue indicators refer to the modifications and the new expressions for rules. To answer the needed updates, in line 3, two additional attributes are defined as well as the attribute ‘countPW’ in line 8 to count the number of failed attempts while entering the password ‘PW’. Another S_t_ entity is instantiated named condition (line 9) to check the subject’s department and role based on (a) and (b) of the above policy updates. Lines 12 to 22 indicate that some ABAC rules are updated to answer the needed modifications in (a). As we can see, an action can be performed on an object if the following are true:
  - The answer of condition (line 17) is true (the condition is true if the value of the dept attribute is equal to that of dept2 or dept3).
  - Another authentication level is verified by entering the correct password ‘PW’ and another condition (contextualCondition) must return true (contextualCondition is true if the value of ‘countPW’ is less than or equal to three).
  Note that the condition is verified at a certain point in time, specifically when something occurs. Hence, this rule deals with the dynamic behavior of the subjects. Note that our grammar is able to express a rule in another way starting with the object. As well, to answer the requirements in (b)-(i) and (b)-(ii), a new rule is expressed in lines 24 to 37.
  (i) An object with attribute type = type-value can be accessed by subjects with attribute address = address-value and perform some action act = act-value1, and some other action act = act-value2 if their dept = dept-value (dept1).
  (ii) An object with attribute type = type-value can be accessed by subjects with attribute address = address-value and perform some action act = act-value1, and some other action act = act-value2 if their dept = dept-value and their role = role-value.

As expressed in Scenarios 1 and 2, we can find that new types of attributes and entities can be defined to describe a larger set of rules to express (then enforce) static and dynamic policies. The above scenarios show the Dynamism of the HEAD metamodel, which comes after its Generality feature.

### 5.3. Extensibility

Developing a generic and dynamic AC metamodel enables developing other important features, such as extensibility. An extensible AC metamodel means that new entities (or attributes) could be defined and integrated with already derived models to support new AC features in addition to the previous ones.

**Figure 20:**
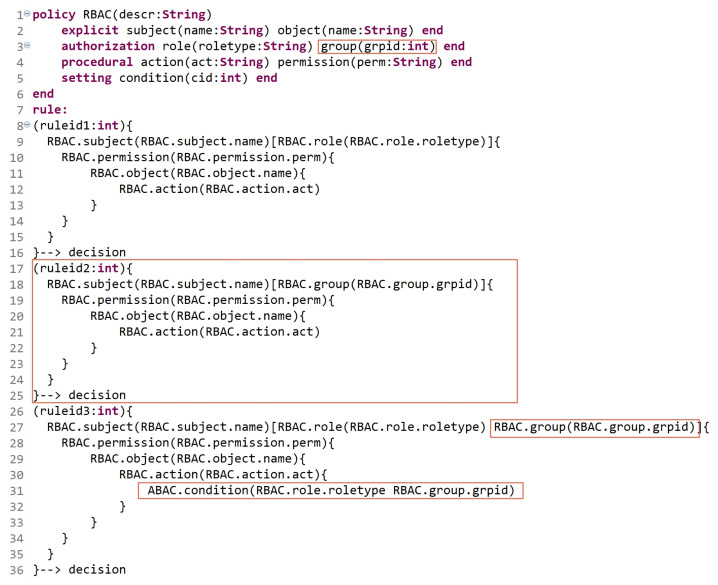
Extensibility: RBAC example.

In this section, we assume that an RBAC model is already defined, and the needed policy is expressed in an organization. Due to new procedures and upgrades in the organization, the users who are already assigned to specific roles need to be classified into groups. For example, subjects who are assigned to role1 are classified into two groups, (group11 and group12), and subjects who are assigned to role2 are classified into three groups, (group21, group22, and group23). Besides the already defined permissions for role1 and role2, other permissions need to be specified based on user-group assignments. Hence, users’ permissions are specified based on their roles, groups, and roles and groups. Figure 20 illustrates an extension for RBAC policy to support the notion of groups. The red indicators show the newly defined entities/attributes in addition to the existing ones with rule expressions. Lines 9 to 18 express the permissions of subjects who are assigned to roles regardless of their groups. Lines 17 to 25 express a new rule of permissions for subjects who are assigned to groups regardless of their roles. Lines 26 to 36 express the permissions of subjects based on their roles and groups; in line 31, conditions are used to check the users’ (subjects’) role and group to allow/deny them performing action(s).

Hence, having an advanced AC metamodel that is able to extend the existing models is a substantial requirement with technology progressions and upgrades.

### 5.4. Hierarchy of Entities

**Figure 21:**
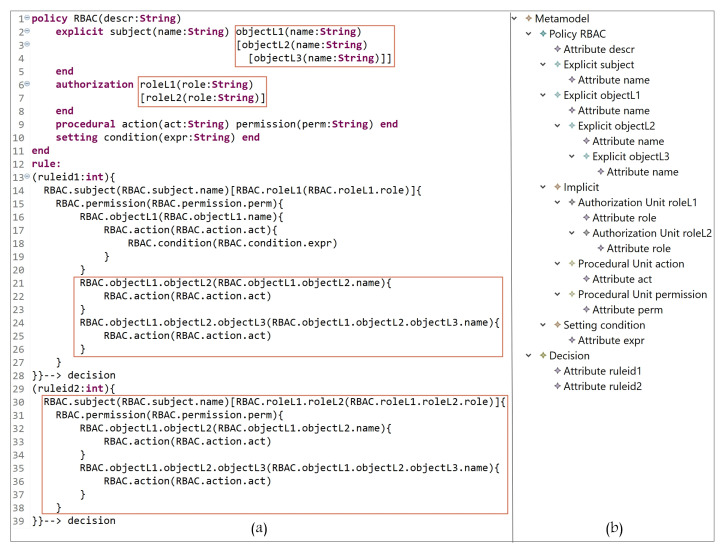
RBAC: (**a**) definition of role/object hierarchy; (**b**) hierarchy of role/object entities.

Hierarchical authorization is the authorization determined based on the hierarchy. Within this structure, access rights are specified by an entity’s place in the hierarchy. The hierarchy defines the relationships between specific types of entities (e.g., roles). This feature can be employed to extend the derived AC models. As we can see in Section 2, several models and metamodels in the literature are extended to support the feature of hierarchy since it provides additional, granular access to resources for an organization and helps reduce maintenance costs. For example, in complex scenarios (e.g., IoT), administrators can start with creating several entities and then add their hierarchy. This would help in managing access to data with less maintenance costs compared to creating a large number of nonhierarchical entities. In this section, we show how HEAD metamodel grammar is able to define a hierarchy for any type of entities. For example, we have the following:

- Creating a hierarchy of roles and objects in RBAC: assume that, after expressing an RBAC policy, an organization needs to update/define new rules that support the hierarchy of roles (two levels of hierarchy) and objects (three levels of hierarchy). In Figure 21a, in lines 2 to 4, we define three levels of object hierarchy: objectL1, objectL2, and objectL3. Note that L1, L2, and L3 are concatenated with the entity name to indicate the level number. In lines 6 to 7, two levels of role are defined (roleL1, and roleL2). In lines 14 to 28, the rule states the following:
  A subject with name = name-value assigned to roleL1 = role-value has permission to access an objectL1 = name-value and perform action act = act-value if condition is true, an objectL2 = name-value and perform action act = act-value, and an objectL3 = name-value and perform action act = act-value.
  In lines 30 to 39, a subject assigned to a role in second level of hierarchy can access an object(s) in a second level of hierarchy, and another object(s) in a third level of hierarchy. The rule states the following:
  A subject with name = name-value assigned to roleL2 = role-value has permission to access an objectL2 = name-value and perform action act = act-value, and an objectL3 = name-value and perform action act = act-value.
  Figure 21b, shows the defined entities/attributes, and the hierarchy of roles and objects. Note that if a subject is assigned to one or more roles, the expression could be written as follows:
  RBAC.subject(RBAC.subject.name)[
    RBAC.roleL1(RBAC.roleL1.role)
    RBAC.roleL1.roleL2(RBAC.roleL1.roleL2.role)
    RBAC.roleL1.roleL2.roleL3(RBAC.roleL1.roleL2.roleL3.role)…]{…

## 6. Generating Policies: Examples and Illustrations

In the previous sections, we defined the grammar of the HEAD metamodel for specifying several AC models, then we defined a model language to describe AC security policies. In this section, the models expressed in the DSL are transformed to Java code in order to generate the AC policies. We provide examples of how the actual AC policies are generated for a given model(s) instances. AC policies are expressed at the system layer, where users interact.

**Figure 22:**
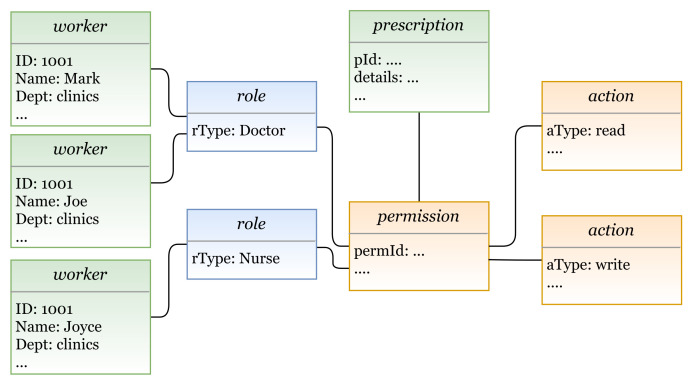
A model instance based on RBAC.

**Figure 23:**
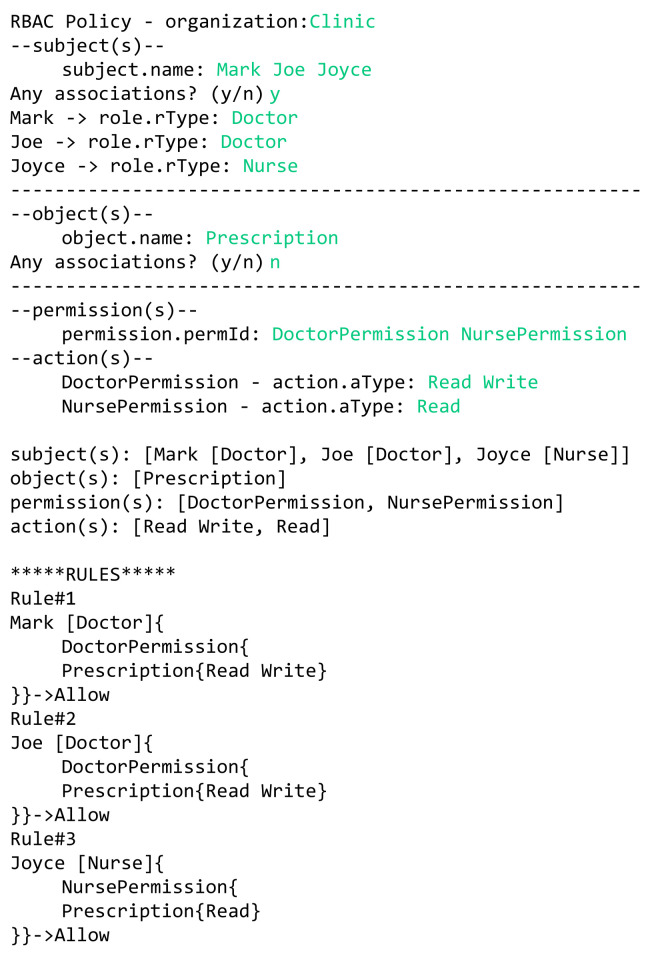
Example 1: generating RBAC policy.

**Figure 24:**
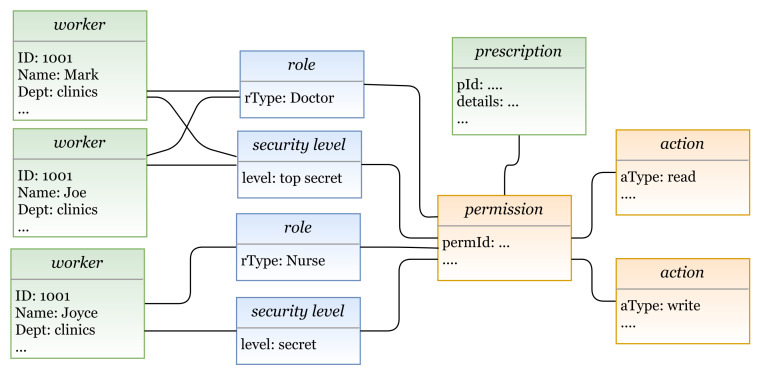
A model instance based on hybrid MAC/RBAC.

**Figure 25:**
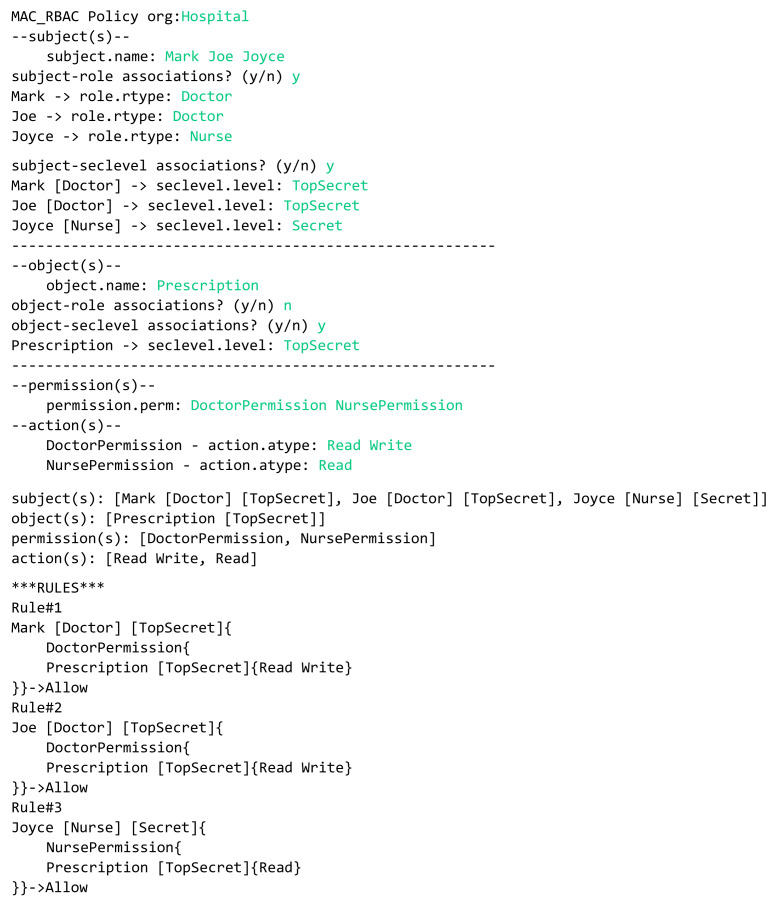
Example 2: Generating MAC/RBAC policy.

- Example 1—RBAC policy: The doctors Mark and Joe in a hospital can read and write patients’ prescriptions. The nurse Joyce is allowed to read these prescriptions.
  In this example we have three rules:
  1. Doctor Mark can read and write patients’ prescriptions.
  2. Doctor Joe can read and write patients’ prescriptions.
  3. The nurse Joyce can read patients’ prescriptions.
  In Figure 22, we illustrate a concrete model instance for the RBAC policy example, which is instantiated from the derived RBAC model based on the HEAD metamodel. For the above policy, we have the following entities/classes:
  - $E_{x}$ entities: subject (worker: name, dept, …), and object (prescription: details, …)
  - AU entities: role (rType, …)
  - PU entities: action (aType, …), permission (permId, …)
  The generated RBAC policy in Figure 23 is modeled based on RBAC model, explained in Section 5.1.3. As shown in the figure, the model includes several elements, and the three rules are generated based on them.
  1. Three subjects (with attribute name): Mark, Joe, and Joyce.
  2. One object (with attribute name): Prescription.
  3. Two roles (with attribute rType): Doctor, and Nurse.
  4. Two permission assignments (with attribute perm): DoctorPermission, and NursePermission.
  5. Two actions (with attribute aType): Read, and Write.
- Example 2—MAC/RBAC policy: In the clinics department of a hospital, the doctors Mark and Joe have a clearance level of “Top Secret” that is equal to the classification level of the object patient prescription. Hence, the doctors are allowed to read/write prescriptions. The nurse Joyce has the clearance level of “Secret” and can read patients’ prescriptions.
  In this example we have the following rules:
  1. Doctor Mark, whose clearance level is “Top Secret”, can read and write prescriptions that have a classification level equal to “Top Secret”.
  2. Doctor Joe, whose clearance level is “Top Secret”, can read and write prescriptions that have a classification level equal to “Top Secret”.
  3. Nurse Joyce, whose clearance level is “Secret”, can only read patients’ prescriptions.
  In Figure 24, we illustrate a concrete model of hybrid MAC/RBAC policy example. Note that, in this example we use BIBA (developed by Kenneth J. Biba) as MAC variant. In the defined policy, some subjects are assigned to doctor and nurse roles. Subjects are permitted to read an object if their clearance level is ⩽ than the object’s classification level, and to write if it is greater than or equal (⩾). Note that if, for example, the clearance level for Doctor Joe is “secret", then he is only allowed to read patients’ prescriptions. Hence, we have the following entities/classes:
  - E${}_{x}$ class(es): subject (worker: name, dept, …), and object (prescription: details)
  - AU class(es): role (rType, …), security level (level, …)
  - PU class(es): action (aType, …), permission (permId, …)
  The generated MAC/RBAC policy in Figure 25 is modeled based on the hybrid MAC/RBAC model, explained in Section 5.1.5. As shown in the figure, the model includes several elements, and the three rules are defined based on them.
  1. Three subjects (with attribute name): Mark, Joe, and Joyce.
  2. One object (with attribute name): Prescription.
  3. Two roles (with attribute rType): Doctor, and Nurse.
  4. Two security levels (with attribute level): Top secret, and Secret.
  5. Two permission assignments (with attribute perm): DoctorPermission, and NursePermission.
  6. Two actions (with attribute aType): Read, and Write.

## 7. Conclusions and Future Perspectives

The evolution of ubiquitous information systems has introduced significant challenges related to security and access control. Information systems should allow users to fulfill transparent access to resources at anytime, anywhere, and in any way, while protecting integrity and confidentiality within the creation of robust security policies. To confront the challenge of accessing resources, various research works were conducted, focusing on developing and enhancing AC modeling in five main directions, starting from (1) traditional access control models, (2) hybrid models, (3) extending AC models, (4) abstracting AC models, reaching to (5) AC metamodels.

On this basis, the objective of this paper is to provide an efficient AC metamodel that conforms to organizational (e.g., companies, industries and hospitals) AC security policies, and adapts the decision making, according to technology progressions to meet organizational and users’ needs. Hence, we propose the HEAD AC metamodel, which takes into consideration the continuous technology changes and upgrades. Its meta-components are constructed after unifying the heterogeneous concepts of AC components. The DSL language of HEAD metamodel is defined for specifying any AC model; it is generic and able to create any component and attribute related to the traditional AC model or any new model. Furthermore, its structure is dynamic and able to define any new component (or attribute) and the relationships between all components; also, any derived model can be extended to follow any technological or organizational updates. Additionally, another powerful feature that exists in the HEAD metamodel is the hierarchy of components (any type of component) to meet hierarchical authorizations. We provide several scenarios to show its generality, dynamism, extensibility, and hierarchy; also, some examples are illustrated to show the generated rules of a policy. Despite providing many advantages, the metamodel may suffer from a drawback, which could be reflected in the vast amount of code that is needed to generate the required AC policies in large and complex systems where all features must be implemented. Nevertheless, several approaches can be implemented to solve this issue.

The emergence of pervasive information systems and intelligent manufacturing has had an extensive impact on different directions, such as the future of the industry. Industry 4.0 is the modernization of traditional manufacturing using modern smart technology. It is based on smart industries where several physical and cyber technologies are merged with the aim of improving productivity, quality, performance, and management in the epoch of IoT. As progressions in technology in general, and IoT in particular, are taking place, the need for security has changed. Hence, organizations and industry sectors have now to rethink how to control access to resources through modern and enhanced AC methods. In Industry 4.0, smart sensors are used to collect huge amounts of environmental data, and a huge number of devices are connected to the internet, from sensors to factory machines, home appliances, hospital tools and equipment, and others. In the field of security and privacy, smart sensors are employed, for example, to send warning alarms to nearby areas in case of fire detection, use facial recognition technologies to send images of a thief to authorities within seconds of theft, and others. As a future perspective, we aim to explain how the HEAD metamodel can be implemented to specify and enforce AC policies, using a detailed case study for an industrial environment (non-IoT and IoT environments), with real applications and results.

## Figures and Tables

**Table 1:** Metamodeling layers and details.

Metamodeling Layers	Details	
	*Description*	*Elements*
Metamodel	Describes the models to be instantiated	explicit, implicit, authorization unit, procedural unit, setting
Model	Metamodel instance, e.g., RBAC model, ABAC model…	subject, object, role, action, permission…
System	Model instance, e.g., RBAC policy, hybrid policy…	Alice, Bob, manager, nurse, prescription, device…

## Data Availability

The study did not report any data.

## Abbreviations

The following abbreviations are used in this manuscript:

AC	Access Control
IoT	Internet of Things
HEAD	Hierarchical, Extensible, Advanced, and Dynamic
DAC	Discretionary Access Control Model
MAC	Mandatory Access Control Model
RBAC	Role-based Access Control Model
ABAC	Attribute-based Access Control Model
IS	Information System
HGABAC	Hierarchical Group and Attribute-based Access Control
HoBAC	Higher-order Attribute-based Access Control
CBAC	Category-based Access Control
CSPM	Cloud Security and Privacy Metamodel
WCMS	Web Content Management System
E${}_{x}$	Explicit
I${}_{m}$	Implicit
AU	Authorization Unit
PU	Procedural Unit
S${}_{t}$	Setting
